# Acupuncture for neurogenesis in experimental ischemic stroke: a systematic review and meta-analysis

**DOI:** 10.1038/srep19521

**Published:** 2016-01-20

**Authors:** Lin Lu, Xiao-guang Zhang, Linda L.D. Zhong, Zi-xian Chen, Yan Li, Guo-qing Zheng, Zhao-xiang Bian

**Affiliations:** 1 School of Chinese Medicine, Hong Kong Baptist University, Hong Kong SAR, China; 2 Department of Neurology, the Second Affiliated Hospital and Yuying Children’s Hospital of Wenzhou Medical University, Wenzhou 325027, China

## Abstract

Acupuncture has been used for patients with stroke and post-stroke rehabilitation for thousands of years. Previous studies reported that acupuncture enhanced stroke recovery through neurogenesis. Hence, we conducted a systematic review and meta-analysis for preclinical studies to assess the current evidence for acupuncture effect on neurogenesis in treating ischaemic stroke. Studies were obtained from six databases, including PubMed, EMBASE, Cochrane Library, Chinese National Knowledge Infrastructure, VIP information database, and Chinese Biomedical Literature Database, Ultimately, 34 studies containing 1617 animals were identified. Neurogenesis markers of Brdu, Nestin, PSA-NCAM, NeuN and GFAP were selected as major outcomes. The pooled results of 15 studies marked with Brdu showed significant effects of acupuncture for improving proliferation when compared with control groups (*P* < 0.01); 13 studies marked with Nestin showed significant effects of acupuncture for increasing proliferation when compared with control groups (*P* < 0.01); 4 studies marked with PSA-NCAM showed significant effects of acupuncture for enhancing migration when compared with control groups (*P* < 0.01); 4 studies marked with NeuN showed significant effects of acupuncture for stimulating differentiation when compared with control groups (*P* < 0.01). The findings suggest that acupuncture is a prospective therapy targeting neurogenesis for ischemic stroke.

Ischemic stroke is one of the leading causes of death and long-term disability worldwide^1^. The only Food and Drug Administration-approved thrombolytic for treating ischemic stroke is recombinant tissue plasminogen activator (rtPA). Unfortunately, rtPA must be administered within 4.5 hours of stroke onset to be effective, and it often results in intracranial hemorrhage^2^. These two factors largely restrict the clinical use of rtPA. Thus, given its widespread occurrence and devastating impact of sufferers and their caregivers, better treatment of ischemic stroke is urgently needed.

Regenerative strategies, particularly with regard to neurogeneisis, offer long-term hope for many patients who have suffered a stroke. Neurogenesis naturally occurs throughout adulthood in the subventricular zone (SVZ) and the subgranular zone (SGZ) of the dentate gyrus (DG) in the hippocampus of the brain. Furthermore, SVZ-derived neural progenitor cells (NPCs) can migrate. When ischemia occurs, genetic fate-mapping studies show that these cells can migrate ectopically into the ischaemic penumbra of the striatum and cerebral cortex^3^, and later, new neurons are generated to replace those lost. This neurogenic response, however, is generally considered too weak to yield enough neurons for compensation and recovery of lost neurocytes and their functions^4^. Instead, there has been some success in supplying neural stem/progenitor cells (NSCs/NPCs) to replace the injured neural cells after acute stroke^5^. Therefore, promoting endogenous neurogenesis appears to be a promising therapeutic strategy for treating stroke, and any treatment that can increase NCSs will test this hypothesis^6^.

Acupuncture is a kind of classical traditional Chinese medicine (TCM) that has been used for patients with stroke and post-stroke rehabilitation for thousands of years^7^. One systematic review of preclinical study indicates that acupuncture decreases infarct volume, ameliorates neurological impairment and plays a neuroprotective role in animal models with acute ischemia^8^. However, the scientific mechanisms of acupuncture for stroke have so far not been fully clarified. Recent reports suggest that acupuncture can enhance stroke recovery through neurogenesis^9^. Therefore, we undertake a preclinical systematic review and meta-analysis to assess the current evidence for acupuncture effect on neurogenesis in treating ischaemic stroke. This review is valuable in several aspects. It can inform the planning and improve the likelihood of success of future clinical trials, provide empirical evidence to improve the rigor of the conducting and reporting of preclinical research, and contribute to both ‘reduction’ and ‘refinement’ in animal experimentation^10^.

## Results

### Study inclusion

**Figure 1:**
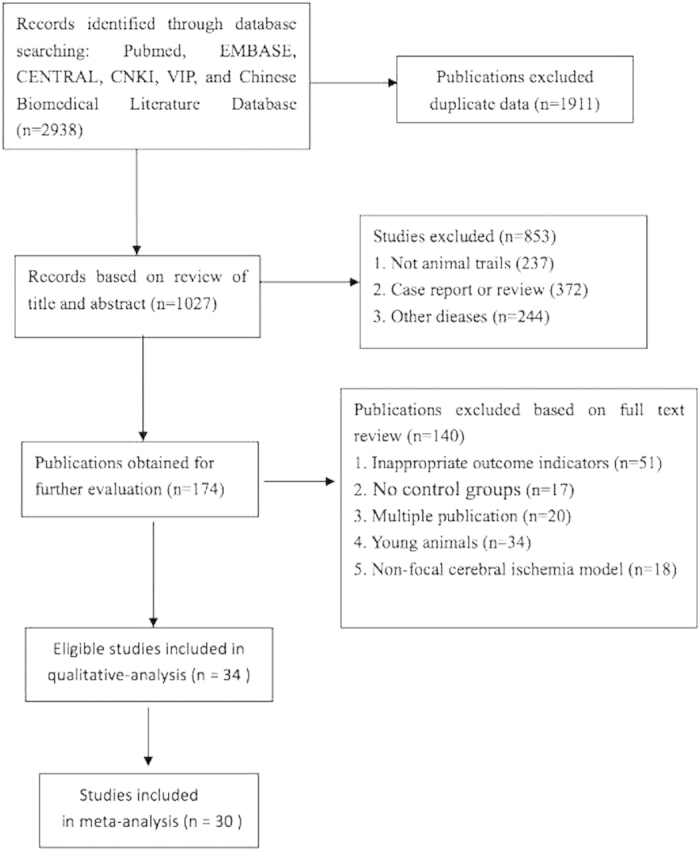
The Preferred Reporting Items for Systematic Reviews and Meta-Analyses (PRISMA) Flow Diagram.

We identified 2938 potentially relevant records from six databases, of which 1027 articles remained after removal of duplicates. After examining the titles and abstracts, 849 papers were excluded for at least one of following reasons: (1) case report or review; (2) not an animal study; (3) not researches study of stroke or ischemia; and (4) immature animals used that were contradiction with neurogenesis. By reading the full text of the remaining 174 articles that reported acupuncture therapy in neurogenesis of experimental ischemic stroke, a total of 140 studies were excluded for the following reasons: 51 used outcome measures marked with none of the neurogenesis indicators, i.e. Brdu, Nestin, PSA-NCAM, NeuN and GFAP; 17 used no control group; 20 were duplicate publication; 34 referred to immature animals; 18 were conducted using a non-focal cerebral ischemia model. Ultimately, 34 eligible studies remained for this systematic review (Fig. 1).

### Study characteristics

The 34 included studies represented a total of 1617 experimental subjects. They described comparisons based on three different kinds of neurogenesis outcome measures^11^,^12^,^13^,^14^,^15^,^16^,^17^,^18^,^19^,^20^,^21^,^22^,^23^,^24^,^25^,^26^,^27^,^28^,^29^,^30^,^31^,^32^,^33^,^34^,^35^,^36^,^37^,^38^,^39^,^40^,^41^,^42^,^43^,^44^: 28 studies reported comparative proliferation data as Brdu and/ or Nestin^11^,^13^,^14^,^15^,^16^,^18^,^19^,^21^,^22^,^23^,^24^,^27^,^28^,^29^,^30^,^31^,^32^,^33^,^34^,^35^,^36^,^37^,^38^,^40^,^41^,^42^,^43^,^44^, 4 studies of comparisons reported migration data as PSA-NCAM^11^,^33^,^34^,^35^, and 15 studies of comparisons reported differentiation data as NeuN and/ or GFAP^11^,^12^,^14^,^17^,^20^,^22^,^24^,^25^,^26^,^32^,^34^,^36^,^39^,^41^,^44^. The animal models used were Sprague-Dawley (SD) rats in 18 studies (52.9%)^12^,^13^,^14^,^18^,^19^,^21^,^22^,^25^,^29^,^30^,^31^,^33^,^34^,^37^,^39^,^41^,^42^,^43^, Wistar rats in 15 studies (44.1%)^11^,^15^,^16^,^17^,^20^,^23^,^24^,^26^,^27^,^28^,^32^,^35^,^36^,^38^,^40^ and C57BL/6 mice in one study (2.9%)^43^. Twenty-three out of the 34 studies (67.6%) utilized temporary MCAO models^11^,^12^,^14^,^15^,^17^,^18^,^19^,^22^,^23^,^24^,^25^,^26^,^27^,^29^,^32^,^33^,^35^,^39^,^40^,^41^,^42^,^43^,^44^, 5 studies (14.7%) utilized permanent MCAO models^13^,^21^,^30^,^34^,^37^, and the remaining studies did not specify^16^,^20^,^28^,^31^,^36^,^38^. To induce anesthesia, 27 studies (78.9%) used chloral hydrate^11^,^12^,^13^,^14^,^15^,^17^,^18^,^19^,^20^,^22^,^23^,^24^,^25^,^26^,^27^,^29^,^30^,^31^,^32^,^33^,^34^,^35^,^36^,^38^,^40^,^42^,^43^, 2 studies used isoflurane (5.9%)^41^,^44^, 3 studies (8.8%) used pentobarbital sodium^21^,^28^,^37^, and 2 studies (5.9%) did not report^16^,^39^. Thirty-three of the included studies (97.1%) adopted electroacupuncture (EA)^11^,^12^,^13^,^14^,^15^,^16^,^17^,^18^,^19^,^20^,^21^,^22^,^23^,^24^,^26^,^27^,^28^,^29^,^30^,^31^,^32^,^33^,^34^,^35^,^36^,^37^,^38^,^39^,^40^,^41^,^42^,^43^,^44^, and 2 (5.9%) used manual acupuncture (MA)^25^,^32^. Neurologic function score (NFS) was reported in 19 studies (55.9%)^11^,^13^,^14^,^15^,^18^,^19^,^21^,^25^,^30^,^31^,^32^,^33^,^34^,^35^,^36^,^37^,^38^,^41^,^43^, infarct size/ volume was reported in 7 studies (20.6%)^19^,^30^,^33^,^34^,^40^,^42^,^43^ and brain water content (BWC) in 2 studies (5.9%)^14^,^39^. Study characteristics were shown in Table 1.

### Study quality

The quality score of the included studies ranged from 3 to 6 out of a total of 10 points. Of the 34 studies, 6 studies (17.6%) got 3 points^16^,^18^,^19^,^20^,^21^,^28^; 9 studies(26.5%) got 4 points^14^,^22^,^24^,^27^,^29^,^32^,^33^,^37^,^39^; 13 studies (38.2%) got 5 points^12^,^15^,^17^,^23^,^25^,^26^,^31^,^35^,^36^,^38^,^40^,^42^,^44^ and 6 studies (17.6%) got 6 points^11^,^13^,^30^,^34^,^41^,^43^ (Table 2). Twenty-seven studies (79.4%) were published in peer review journals^11^,^12^,^13^,^14^,^16^,^17^,^18^,^19^,^20^,^21^,^22^,^23^,^24^,^25^,^26^,^27^,^28^,^34^,^35^, and 7 studies (20.6%) were online master’s theses or PhD theses with not formally published^15^,^29^,^30^,^31^,^32^,^33^,^36^. Nineteen studies (55.9%) described control of the room temperature^11^,^12^,^15^,^22^,^23^,^25^,^26^,^29^,^30^,^31^,^32^,^33^,^34^,^35^,^36^,^38^,^41^,^43^,^44^. Hyperlipemia^13^ and aged^14^ rats were used in each of two studies (2.9%, respectively), and hypertensive rats were used in two studies (5.8%)^30^,^34^. Thirty-two studies (94.1%) reported random allocation to treatment group^11^,^12^,^13^,^15^,^43^. Only one study (2.9%) described masked induction of stroke model^16^. For two studies (5.8%) it could not be certified if the anesthetic used had significant intrinsic neuroprotective activity^14^,^16^. Twenty-four studies (70.6%) mentioned compliance with animal welfare regulations^11^,^12^,^13^,^14^,^15^,^17^,^23^,^24^,^25^,^26^,^27^,^29^,^30^,^31^,^35^,^36^,^37^,^38^,^39^,^40^,^41^,^42^,^43^,^44^. Sixteen studies (47.1%) contained statements of potential conflict of interests^11^,^13^,^14^,^15^,^17^,^30^,^31^,^32^,^33^,^34^,^36^,^40^,^41^,^42^,^43^,^44^. None of the studies reported blinded assessment of outcome or sample size calculation.

### Effectiveness

#### Neurological function score

**Figure 2:**
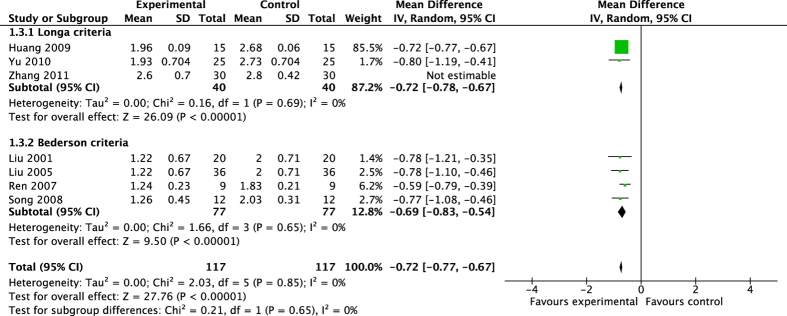
Pooled result of neurological function score based on acupuncture therapy in experimental ischemic stroke.

Six studies including 234 rats were conducted the meta-analysis of neurological function score^11^,^13^,^21^,^25^,^37^,^38^. The pooled results (Fig. 2) showed that acupuncture significantly ameliorated neurological deficiency after ischemic injury by either Longa criterion or Bederson criterion (Longa criterion: MD −0.72, 95% CI [−0.78, −0.67], *P* < 0.00001; Heterogeneity: Chi^2^ = 0.16, df = 1 (P = 0.69), I^2^ = 0%. Bederson criterion: MD −0.69, 95%CI [−0.83, −0.54], *P* < 0.00001; Heterogeneity: Chi^2^ = 1.66, df = 3 (P = 0.65); I^2^ = 0%). The overall effect of acupuncture on neurological function by different criteria was MD −0.72, 95% CI [−0.77, −0.67], *P* < 0.00001; Heterogeneity: Chi^2^ = 2.03, df = 5 (P = 0.85), I^2^ = 0%. The study^35^ was not estimated in the sub-analysis of Longa criterion because of leading to low homogeneous result (Heterogeneity: Chi^2^ = 12.00, df = 2 (P = 0.002), I^2^ = 83%) whereas decreased effect size by 0.14 yielding a still significant pooled MD −0.58, 95%CI [−0.92, −0.23], *P* = 0.001, which was similar with previous analysis (MD −0.58 vs MD −0.72).

#### Brain water content (BWC) and infarct volume (IV)

Two studies included 24 rats were conducted pooled analysis of BWC^14^,^39^. The pooled results showed that acupuncture significantly decreased BWC (MD −3.03, 95% CI [−4.42, −1.63], *P* < 0.0001; Heterogeneity: Chi^2^ = 5.52, df = 1 (P = 0.02), I^2^ = 82%). Pooled results of two studies^30^,^33^ showed that acupuncture had no significant effect on IV (MD −4.43, 95% CI [−11.79, 2.94], *P* = 0.24; Heterogeneity: Chi^2^ = 0.15, df = 1 (P = 0.70), I^2^ = 0%).

### Overall effect of acupuncture on neurogenesis

#### Proliferation: Brdu^+^ cells and Nestin^+^ cells

**Figure 3:**
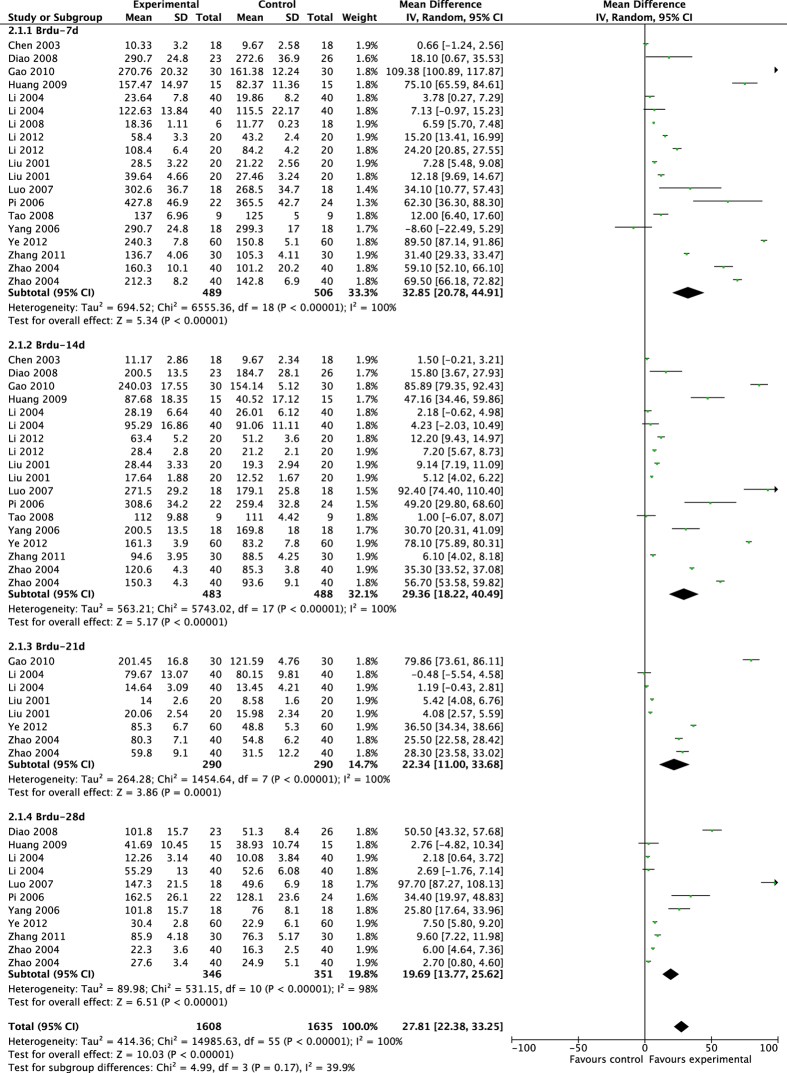
Pooled result of Brdu^+^ cells based on acupuncture therapy in experimental ischemic stroke.

**Figure 4:**
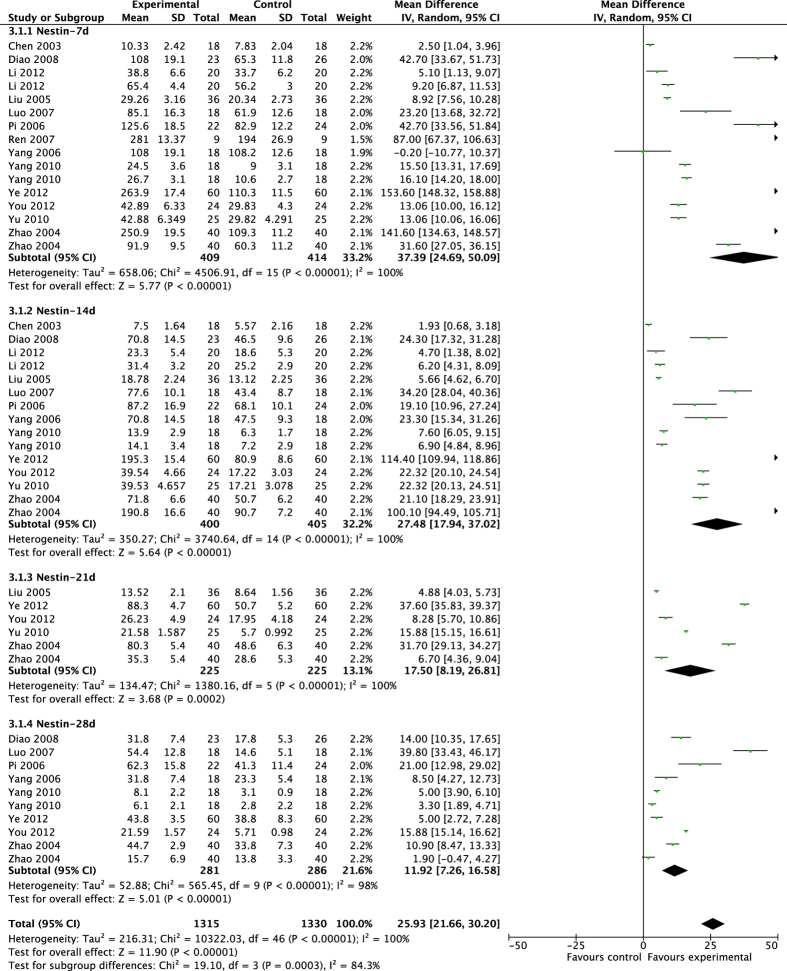
Pooled result of Nestin^+^ cells based on acupuncture therapy in experimental ischemic stroke.

The pooled results showed that acupuncture significantly enhanced proliferation after brain ischemia (Figs 3 and 4). From day 7 to day 28 after MCAO surgery, acupuncture significantly increased the expression of Brdu^+^ cells and Nestin^+^ cells compared with control groups at each time of point (Brdu-7d: MD 32.85, 95% CI [20.78, 44.91], *P* < 0.00001; Heterogeneity: Chi^2^ = 6555.36, df = 18 (P < 0.00001), I^2^ = 100%; Brdu-14d: MD 29.36, 95%CI [18.22, 40.49], *P* < 0.00001; Heterogeneity: Chi^2^ = 5743.02, df = 17 (P < 0.00001), I^2^ = 100%; Brdu-21d: MD 22.34, 95% CI [11.00, 33.68], *P = *0.0001; Heterogeneity: Chi^2^ = 1454.64, df = 7 (P < 0.00001), I^2^ = 100%; Brdu-28d: MD 19.69, 95% CI [13.77, 25.62], *P* < 0.00001; Heterogeneity: Chi^2^ = 531.15, df = 10 (P < 0.00001), I^2^ = 98%; Overall effect: MD 27.81, 95% CI [22.38, 33.25], *P* < 0.00001; Heterogeneity: Chi^2^ = 14985.63, df = 55 (P < 0.00001), I^2^ = 100%. Nestin-7d: MD 37.39, 95% CI [24.69, 50.09], *P* < 0.00001; Heterogeneity: Chi^2^ = 4506.91, df = 15 (P < 0.00001), I^2^ = 100%; Nestin-14d: MD 27.48, 95% CI [17.94, 37.02], *P* < 0.00001; Heterogeneity: Chi^2^ = 3740.64, df = 14 (P < 0.00001), I^2^ = 100%; Nestin-21d: MD 17.50, 95% CI [8.19, 26.81], *P* = 0.0002; Heterogeneity: Chi^2^ = 1380.16, df = 5 (P < 0.00001), I^2^ = 100%; Nestin-28d: MD 11.92, 95% CI [7.26, 16.58], *P* < 0.00001; Heterogeneity: Chi^2^ = 565.45, df = 9 (P < 0.00001), I^2^ = 98%; Overall effect: MD 25.93, 95% CI [21.66, 30.20], *P* < 0.00001;Heterogeneity: Chi^2^ = 10322.03, df = 46 (P < 0.00001), I^2^ = 100%).

#### Migration: PSA-NCAM^+^ cells

**Figure 5:**
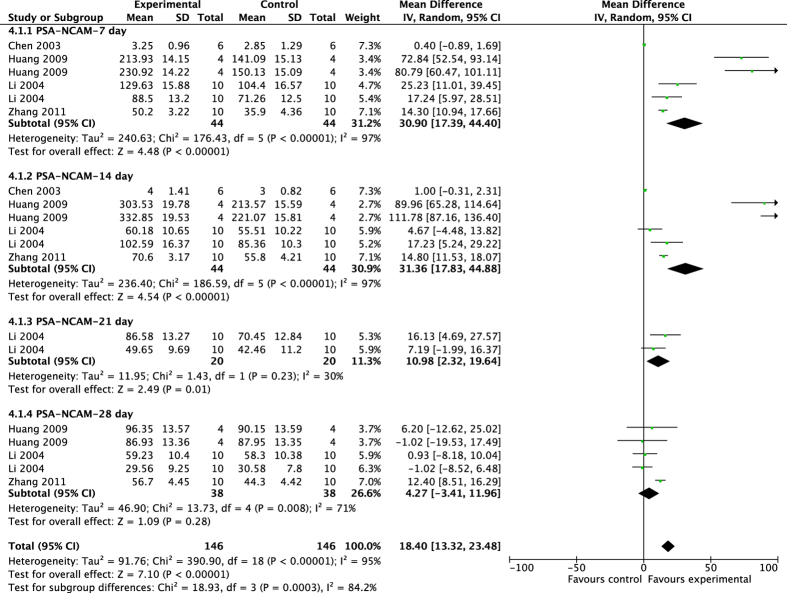
Pooled result of PSA-NCAM^+^ cells based on acupuncture therapy in experimental ischemic stroke.

The pooled results showed that acupuncture significantly improved migrating process after ischemic injury (Fig. 5), with higher expression of PSA-NCAM^+^ cells compared with control groups (7d: MD 30.90, 95% CI [17.39, 44.40], *P* < 0.00001; Heterogeneity: Chi^2^ = 176.43, df = 5 (P < 0.00001), I^2^ = 97%; 14d: MD 31.36, 95% CI [17.83, 44.88], *P* < 0.0001; Heterogeneity: Chi^2^ = 10.64, df = 4 (P = 0.03), I^2^ = 62%; 21d: MD 10.98, 95% CI [2.32, 19.64], *P* = 0.01; Heterogeneity: Chi^2^ = 1.43, df = 1 (P = 0.23), I^2^ = 30%; 28d: MD 4.27, 95% CI [−3.41, 11.96], *P* = 0.28; Heterogeneity: Chi^2^ = 13.73, df = 4 (P = 0.008), I^2^ = 71%; Overall effect: MD 18.40, 95% CI [13.32, 23.48], *P* < 0.0001; Heterogeneity: Chi^2^ = 390.90, df = 18 (P < 0.00001), I^2^ = 95%.

#### Differentiation: NeuN^+^ cells and GFAP^+^ cells

**Figure 6:**
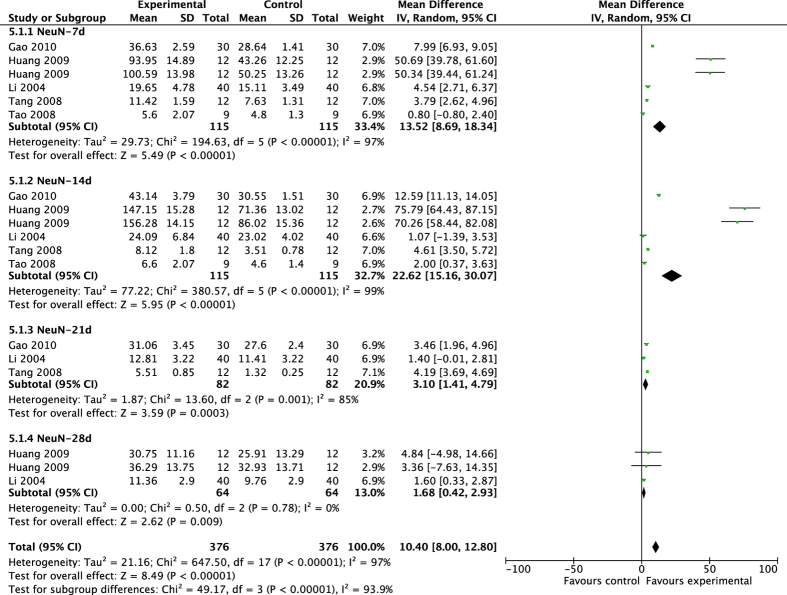
Pooled result of NeuN^+^ cells based on acupuncture therapy in experimental ischemic stroke.

**Figure 7:**
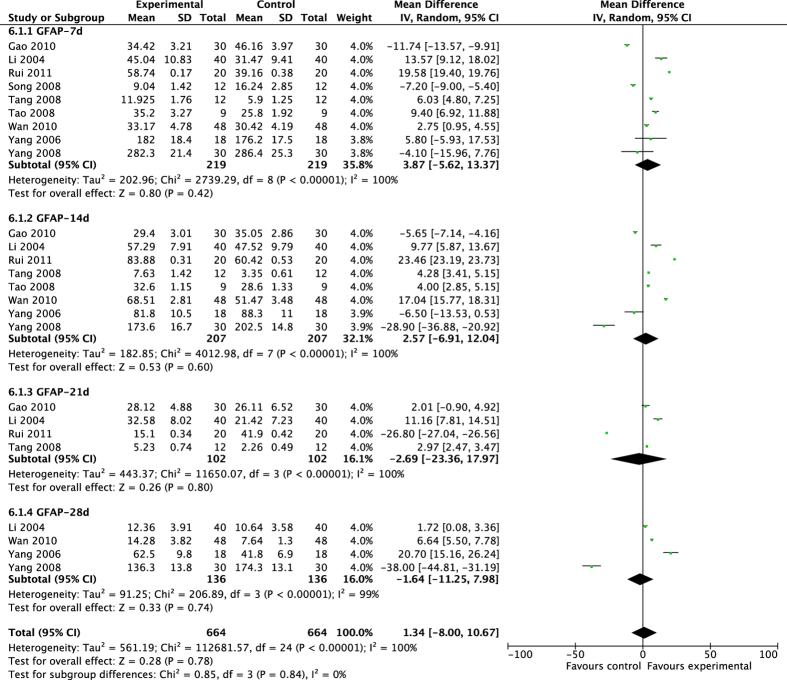
Pooled result of GFAP^+^ cells based on acupuncture therapy in experimental ischemic stroke.

The pooled results showed that acupuncture significantly increased neuron differentiation, other than gliocyte differentiation (Figs 6 and 7). Acupuncture significantly improved the expression of NeuN^+^ cells, but had no noticeable effect on GFAP^+^ cells in MCAO rats (NeuN-7d: MD 13.52, 95% CI [8.69, 18.34], *P* < 0.00001; Heterogeneity: Chi^2^ = 194.63, df = 5 (P < 0.00001), I^2^ = 97%; NeuN-14d: MD 22.62, 95% CI [15.16, 30.07], *P* < 0.00001; Heterogeneity: Chi^2^ = 380.57, df = 5 (P < 0.00001), I^2^ = 99%; NeuN-21d: MD 3.10, 95% CI [1.41, 4.79], *P* = 0.0003; Heterogeneity: Chi^2^ = 13.60, df = 2 (P = 0.001), I^2^ = 85%; NeuN-28d: MD 1.68, 95% CI [0.42, 2.93], *P* = 0.009; Heterogeneity: Chi^2^ = 0.50, df = 2 (P = 0.78), I^2^ = 0%; Overall effect: MD 10.40, 95% CI [8.00, 12.80], *P* < 0.00001; Heterogeneity: Chi^2^ = 647.50, df = 17 (P < 0.00001), I^2^ = 97%. GFAP-7d: MD 3.87 [−5.62, 13.37], *P* = 0.42; Heterogeneity: Chi^2^ = 2739.29, df = 8 (P < 0.00001), I^2^ = 100%; GFAP-14d: MD 2.57, 95% CI [−6.91, 12.04], *P* = 0.60; Heterogeneity: Chi^2^ = 4012.98, df = 7 (P < 0.00001); I^2^ = 100%; GFAP-21d: MD −2.69, 95% CI [−23.36, 17.97], *P* = 0.80; Heterogeneity: Chi^2^ = 11650.07, df = 3 (P < 0.00001), I^2^ = 100%; GFAP-28d: MD −1.64, 95% CI [−11.25, 7.98], *P* = 0.74; Heterogeneity: Chi^2^ = 206.89, df = 3 (P < 0.00001), I^2^ = 99%; Overall effect: MD 1.34, 95% CI [−8.00, 10.67], *P* = 0.78; Heterogeneity: Chi^2^ = 112681.57, df = 24 (P < 0.00001), I^2^ = 100%).

Four studies in English^41^,^42^,^43^,^44^, which did not conduct quantitative analyses, all reported that acupuncture had positive significant effects on neurogenesis particularly on proliferation of neurocytes (*P* < 0.05). Among the four studies, neurobehavioral change was significantly improved, and infarct volume was noticeably declined in two studies^41^,^42^.

### Subgroup analyses and sensitivity analyses

To explore other factors which potentially affected the outcome measures, we stratified the included studies according to variables as shown in Table 3. During the course of neurogenesis, the differentiation process chronologically depended on mature proliferation and migration. Based on the above results of overall effects for acupuncture on neurogenesis, we conducted the subgroup analyses and sensitivity analyses of Brdu, Nestin, and PSA-NCAM markers based on the time point of 7 days after ischemic injury, and of NeuN marker at 14 days after ischemia. However, no subgroup analysis and sensitivity analysis of GFAP marker were conducted at any time point as the pooled result of the overall effect did not have significant difference. (1) For Brdu^+^ cells, the stratified analysis showed that significant differences in effect sizes were observed relative to the brain site of the target detection (*P* < 0.001), animal species (*P* = 0.01), the type of MCAO model (*P* < 0.001), anesthetic used (P < 0.001), duration of treatment (*P* < 0.001), and the baseline median of positive cells (*P* = 0.01). No significant differences in effect sizes was observed relative to animal weight (*P* = 0.79). (2) For Nestin^+^ cells, the stratified analysis showed that significant differences in effect sizes were observed relative to the brain site of the target detection (*P* < 0.001), animal species (*P* = 0.006), anesthetic used (*P* < 0.001), animal weight (*P* = 0.001), duration of treatment (*P* < 0.001), and the baseline median of positive cells (*P* = 0.02). No significant differences in effect sizes was observed relative to the type of MCAO model (*P* = 0.14). (3) For PSA-NCAM^+^ cells, the stratified analysis showed that significant differences in effect sizes were observed relative to animal species (*P* < 0.001), the type of MCAO model (*P* < 0.001), and the baseline median of positive cells (*P* = 0.01). No significant differences in effect sizes were observed relative to the brain site of the target detection (*P* = 0.15), and animal weight (*P* = 0.11). (4) For NeuN^+^ cells, the stratified analysis showed that significant differences in effect sizes were observed relative to the brain site of the target detection (*P* < 0.001), the type of MCAO model (*P* < 0.001), animal weight (*P* < 0.001), and duration of treatment (*P* < 0.001). No significant differences in effect sizes was observed relative to animal species (*P* = 0.22).

Sensitivity analyses showed that the results did not substantially alter after removing any one trial. Furthermore, respectively excluding the one in the three studies^11^,^13^,^27^ that contributed least to the overall estimate of each marker also did not materially alter the results (Brdu: MD 26.89, 95%CI [21.33, 32.45], *P* < 0.00001; Nesstin: MD 25.50, 95%CI [21.10, 29.90], *P* < 0.00001; PSA-NCAM: MD 1.28, 95%CI [0.37, 2.19], *P* < 0.006; NeuN: MD 4.03, 95%CI [3.26, 4.80], *P* < 0.00001).

### Meta-regression

To further explore heterogeneity among the studies, meta-regression was conducted to investigate the effect of characteristics on the positive markers (Table 3). (1) For Brdu^+^ cells, we found that animal species (Adjusted R^2^ = 14.33%), the type of MCAO model (Adjusted R^2^ = 23.17%), duration of treatment (Adjusted R^2^ = 31.16%), and the baseline median of positive cells (Adjusted R^2^ = 24.43%) accounted for a significant proportion of the between-study heterogeneity in studies. (2) For Nestin^+^ cells, anesthetic used (Adjusted R^2^ = 6.22%), duration of treatment (Adjusted R^2^ = 7.70%), and the baseline median of positive cells (Adjusted R^2^ = 48.78%) were significant sources of heterogeneity. Meta-regression was not conducted in PSA-NCAM, NeuN and GFAP markers as less than 10 studies were included in each outcome measure.

### Assessment of publication bias

**Figure 8:**
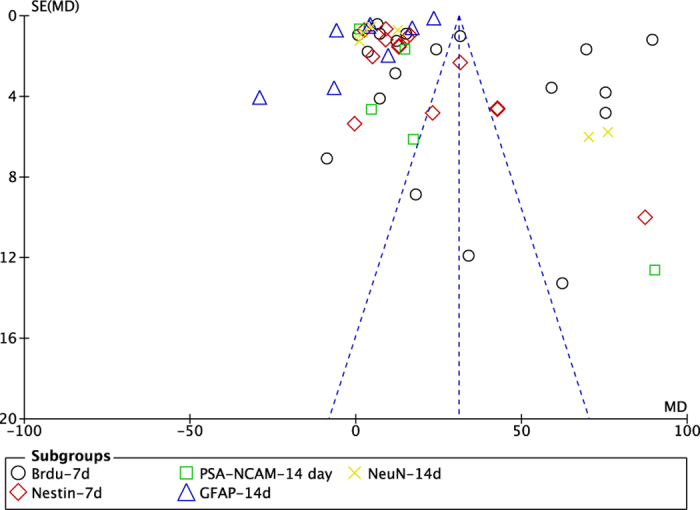
The funnel plot of the neurogenesis effect of acupuncture after experimental ischemic stroke.

Funnel plot (Fig. 8) showed asymmetry, indicating a potential publication bias. Through Egger’s test, the publication existed in Brdu, Nestin, PSA-NCAM and NeuN data (p = 0.000, p = 0.000, p = 0.005 and p = 0.002, respectively), Figure S1–S4. There was no significant bias in GFAP data with Egger’s test (p = 0.442), Figure S5.

## Discussion

### Efficacy of acupuncture

This systematic review found that acupuncture improved neurological deficits and reduced brain edema in experimental ischemia, and that the mechanisms mostly involved with enhancing endogenous neurogenesis including proliferation, migration and differentiation of NSCs.

### Methodological considerations

There were some limitations to consider in interpreting our study. Firstly, we were unable to meta-analyze all trials due to insufficient data in several primary studies included^41^,^42^,^43^,^44^. Although they all claimed positive effects of acupuncture on neurogenesis after ischemia. Secondly, publication bias existed by the asymmetry of the funnel plot and statistical analysis with Egger’s test. Some non-positive studies have been missed inevitability, as negative findings are less likely to be published^45^. Publication bias inflated estimates by a flawed methodological design in smaller studies, and/or a lack of publication of small trials with opposite results. Thus, the overall positive findings of treatment with acupuncture should made with caution. Thirdly, the qualitative score ranging from 3 to 6 points indicated poor methodological quality in the included studies. Randomization, blinding, sample-size estimation and the handling of all data should be reported clearly, as these are the core standards of rigorous study design^46^. Although 32 studies described random allocation to a treatment group, none of them reported details of how the animals were randomized. Only one study described masked induction of stroke model. None of the included studies reported the sample-size calculation or whether investigators were blind to the outcome. Low quality of the included studies suggested that the results should be interpreted with caution.

### Heterogeneity interpretation

The heterogeneity to some extent existed in the meta-analyses of neurogenesis that represented with five specific biological markers, i.e. Brdu, Nestin, PSA-NCAM, NeuN and GFAP. According to the sub-analyses and meta-regression, however, a significant linear relation was demonstrated between the brain site of neurogenesis and acupuncture therapy, animal species, type of MCAO model, anesthetic, duration of treatment and the baseline median of positive cells. In addition, duration of treatment and the baseline median of positive cells were the most significant proportion of the between-study heterogeneity in studies. Higher level of median baseline accounted for greater beneficial effect, which would contributed to different trials adopted various counting methods to calculate the positive cells, as some studies counted cells under microscope with different folds, visual fields and slices of sample, or even with unequal sizes of interested areas. Also, either optical density or grayscale was used in image analysis.

### Implication for further studies

In the present study, acupuncture was found to be effective in neurogenesis, particularly from day 7 to day 14 after ischemia; neurocyte proliferation peaked at day 7 while differentiation peaked at day 14. These results suggested an optimum time window in stroke for acupuncture therapy. Meanwhile, we found that the most used acupuncture points were Baihui (GV 20), Zusanli (ST 36), Dazhui (GV 14) and Quchi (LI 11). They were used individually or in combination. Based on TCM theory, Baihui (GV 20) is located on the highest place of the head where all the yang meridians meet^47^. Acupuncture on Baihui (GV 20) is prefer to clear the mind, lift the spirits, tonify yang, strengthen the ascending function of the spleen, eliminate interior wind, and promote resuscitation^48^. Thus, Baihui (GV 20) is a principle acupoint which is often selected for stroke patients.

After all, several included studies speculated on how acupuncture enhanced neurogenesis, and proposed the following possible biological mechanisms: up-regulating the expression of the signaling molecules of the PKA-CREB transduction system in the hippocampus of the ipsilateral hemispheres^11^, enhancing the brain-derived neurotrophic factor (BDNF) and vascular endothelial growth factor (VEGF) signaling pathways^44^, improving the levels of neurotrophic factors BDNF, NGF, bFGF, NSE and FGF-2 13,31,32,34,36,40, activating the ERK signal transduction pathway^15^, and up-regulating the expression of GAP-43, TrkB and down-regulating the expression of Rho Kinase^31^. Thus, we suggest that the endogenous neurogenesis after ischemia should be further performed with their physiological function by electrophysiology and other methodologies. Meanwhile, future well-designed studies are warranted to fully clarify the mechanisms of acupuncture inducing endogenous neurogenesis.

## Conclusion

Our findings indicate that acupuncture ameliorates neurological deficits and reduces brain edema in experimental ischemic stroke, and the mechanisms positively correlate with endogenous neurogenesis, in which acupuncture therapy can promote the proliferation, migration and differentiation of NSCs.

## Methods

### Search strategy

Studies of acupuncture on neurogenesis treatment after experimental ischemic stroke models were derived from PubMed, EMBASE, Cochrane Library, Chinese National Knowledge Infrastructure (CNKI), VIP information database, and Chinese Biomedical Literature Database. We also manually searched abstracts of academic seminars and reference lists of identified publications, and we contacted researchers to retrieve unpublished and/or unreported materials that might be relevant. All searches were performed from the establishment of each database up to August 2015. Database searches were conducted using the following terms: (acupuncture OR electroacupuncture (EA) OR moxibustion) AND (stroke OR cerebral ischemia) AND (neurogenesis OR neural regeneration OR neurotization). Chinese Databases were also searched using the above search terms in Chinese. All searches were limited to studies on animals.

### Eligibility

We included all identified studies of acupuncture in neurogenesis of experimental ischemic stroke where the outcome was measured as bromodeoxyuridine (Brdu) and/or Nestin and/or polysialylated form of the neural cell adhesion molecule (PSA-NCAM) and/or neuronal nuclear antigen (NeuN) and/or glial fibrillary acidic protein (GFAP). Neurological function score (NFS) or/ and infarct volume (IV) or/ and brain water content (BWC) were also extracted as outcome indicators, as appropriate. Brdu, being used for monitoring cell proliferation, is a synthetic thymidine analog that incorporates DNA of dividing cells during the S-phase of the cell cycle. Nestin is defined as a class VI intermediate filament protein that is known as a NSC/ NPC marker during development of the central nervous system (CNS). Polysialic acid (PSA) is a linear homopolymer of alpha2–8-N acetylneuraminic acid whose primary vector in vertebrates is the neural cell adhesion molecule (NCAM). PSA-NCAM participates in neural plasticity and neurogenesis and it is particularly considered towards cell migration. NeuN, a DNA-binding protein that is abundantly and exclusively expressed in neurons of the CNS, is considered to be a hallmark of neuronal differentiation during development. Not only do neurons immunolabeled NeuN differentiate from gliocyte and endothelial cells, but also NeuN identifies morphologically distinct classes of neurons in brain structures. GFAP is an intermediate-filament protein expressed uniquely in astrocytes and vulnerable to reactive gliosis that follows injuries to the CNS. It is described as one of the labels of astrocytic differentiation in vertebrates^53^,^54^. NFS, IV and BWC indicators are also known to contribute to the efficacy of acupuncture in neurogenesis of ischemic stroke^8^.

Inclusion criteria were as follows: (1) studies of experimental ischemic stroke induced by temporary middle cerebral artery occlusion (MCAO) or permanent MCAO; (2) randomized studies of manual acupuncture (MA) or electroacupuncture (EA); and (3) studies using at least one of the following neurogenesis indexes Brdu, Nestin, PSA-NCAM, NeuN and GFAP as outcome measures; (4) studies results comparing experimental groups with control groups receiving placebo/ sham acupuncture or no treatment. Exclusion criteria were that the studies used a non-focal cerebral ischemia model (such as global, traumatic models, or hypoxic-ischemic models), that they used young animals, that they had no control group, or that the same study was published in duplicate.

### Data extraction

The following details were extracted from each study: (1) publication year and the first author’s name, type of ischemic stroke (transient, or permanent), and ischemic time; (2) animal model details, namely species, sex, weight and number of animals per group; (3) treatment information, including timing for initial treatment, types and methods of treatment, duration of treatment, and comparable treatment of control group; (4) outcome measure information, including the phase of neurogenesis, outcome indexes, timing for outcomes assessments and the corresponding *p* values. (5) For each comparison, we extracted data of mean value and standard deviation from each treatment and control group of each study. If the data for meta-analysis were missing or only expressed graphically, we tried to contact the authors for the missing, or more specific information. Otherwise we only performed qualitative analysis.

### Quality assessment

Two authors independently assessed the methodological quality of the included articles according to the CAMARADES 10-item checklist^55^: (1) peer-reviewed publication; (2) statements of temperature control; (3) randomization to treatment or control group; (4) blinded induction of model; (5) blinded assessment of outcome; (6) use of anesthetic without significant intrinsic neuroprotective activity; (7) appropriate animal model; (8) sample size calculation; (9) compliance with animal welfare regulations; and (10) declaration of potential conflict of interests. Each study was given an aggregate quality score out of a possible total of 10 points, and the group median was calculated. Discrepancies were resolved after discussion between the two authors or were referred to an arbitrator.

### Statistical analysis

Meta-analyses and sub-analyses were performed using RevMan V.5.1 software, and analyses of public bias and meta-regression were performed using STATA/SE 12.0 software. Outcome indicators of Brdu, Nestin, PSA-NCAM, NeuN, GFAP, NFS, IV, and BWC were all considered as continuous data, and these indicators conducted an estimate of the combined effect sizes utilizing mean difference (MD) with the random effects model, respectively. Publication bias was assessed with a funnel plot and Egger’s test^56^. The I^2^ statistic was used for assessment of heterogeneity. To clarify the impact of factors potentially modifying the outcome measures, we also conducted sensitivity analyses and subgroup analyses according to the following variables: encephalic region of neurogenesis, animal species, anaesthetic used, MCAO model induction, acupuncture method used, and baseline median of positive cells. Difference between n groups was assessed by partitioning heterogeneity and using the χ^2^ distribution with n-1 degrees of freedom (df), where n meant the number of groups.

## Additional Information

**How to cite this article**: Lu, L. *et al.* Acupuncture for neurogenesis in experimental ischemic stroke: a systematic review and meta-analysis. *Sci. Rep.* **6**, 19521; doi: 10.1038/srep19521 (2016).

## Supplementary Material

Supplementary Information

## Figures and Tables

**Table 1:** Characteristics of the 34 included studies.

Study (years)	Species (Sex, n = experimental /control group )	Weight	Model (method)	Anesthetic	Treatment group (Method to acupuncture)	Control group	Neurogenesis	Outcome Index (time)	Intergroup Differences
Huang 2009 (11)	Wistar rats (male, 15/15)	200 ± 20 g	MCAO/ 2 h	6% chloralhydrate (35 mg/100 g)	EA 30 min after reperfusion per day, with frequency of 20 Hz and current density of 1–2 mA (baihui, dazhui)	Non-EA	Proliferation, migration, differentiation	1. NFS (ZL, 7, 14, 28d); 2. Brdu^+^ cells; 3. PSA-NCAM^+^ cells; 4. Brdu/NeuN^+^ cells.	1. *P* < 0.01; 2. *P* < 0.05 (7, 14d), *P* > 0.05 (28d); 3. *P* < 0.05 (7, 14d), *P* > 0.05 (28d); 4. *P* < 0.05 (7, 14d), *P* > 0.05 (28d).
Yan 2008 (12)	SD rats (male, 12/12)	200–250 g	MCAO/30 min	10% chloralhydrate (0.3 ml/100 g)	EA 5 min after reperfusion per day (shuigou, neiguan, quchi, zusanli)	Non-EA	Differentiation	1. CyclinD1 level (2d, 7d); 2. GFAP level	1. *P* < 0.05, *P* > 0.05; 2. *P* < 0.05, *P* < 0.01
Ren 2007 (13)	SD rats (male, 9/9)	200 ± 20 g	PermanentMCAO (Tamura)	10% chloralhydrate (3.5 ml/1 Kg)	EA 20 min after surgery per day, with frequency of 15 Hz and current density of 1–3 mA (baihui, renzhong)	Non-EA	Proliferation, differentiation	1. NFS (Bederson, 7d); 2. Nestin^+^ cells; 3. PNCA^+^ cells; 4. Vimentin^+^ cells; 5. Tuj-1^+^ cells;	1. *P* < 0.05; 2. *P* < 0.01; 3. *P* < 0.01; 4. *P* < 0.01; 5. *P* < 0.01.
Gao 2010 (14)	SD rats (male, 30/30)	450–600 g	MCAO/3 h (ZL)	10% chloralhydrate (0.3 ml/100 g)	EA 10 min after reperfusion at once, 1 h, 2 h and then per day (jianyu, biguan, zusanli, waiguan)	Non-EA	Proliferation, differentiation	1. NFS (1, 3, 7, 14, 21d); 2. BWC; 3. Brdu^+^ cells; 4. Brdu/NeuN^+^ cells; 5.Brdu/GFAP^+^ cells;	1. *P* < 0.01 (3, 7, 14, 21d); 2. *P* < 0.01 (1, 3, 7d); 3. *P* < 0.01; 4. *P* < 0.01; 5. *P* < 0.05 (3, 7, 14d);
Luo 2007 (15)	Wistar rats (male, 18/18)	250 ± 10 g	MCAO/2 h	10% chloralhydrate (35 mg/100 g)	EA 20 min after reperfusion per day, with frequency of 30/100 Hz and current density of 6–15 V (Du: renzhong, baihui)	Non-EA	Proliferation	1. NFS (ZL, 7, 14, 28d); 2. Brdu^+^ cells; 3. Brdu/Nestin^+^ cells	1. *P* < 0.01; 2. *P* < 0.01 (14, 28d), *P* > 0.05 (7d); 3. *P* < 0.01 (14, 28d), *P* > 0.05 (7d);
					(Rendu: chengjiang, shenyu, guanyuan, renzhong, baihui)			2. Brdu^+^ cells; 3. Brdu/Nestin^+^ cells	2. *P* < 0.01; 3. *P* < 0.01
Yang 2010 (16)	Wistar rats (male, 18/18)	NR	MCAO	NR	EA 10 min after surgery per day (guanyuan, qihai, chengjiang)	Non-EA	Proliferation	1. EGFPP/Nestin^+^ cells (7, 14, 28d)	1. *P* < 0.01
Rui 2011 (17)	Wistar rats (male, 20/20)	250 ± 30 g	MCAO/2 h (ZL)	10% chloralhydrate (0.3 ml/100 g)	EA 30 min after reperfusion per day, with frequency of 10 HZ and current density of 3–5 mA (baihui, fengfu)	Non-EA	Differentiation	1. Brdu/NSE^+^ cells (3, 7, 14, 21d); 2. Brdu/GFAP^+^ cells	1. *P* < 0.01; 2. *P* < 0.01.
Li 2012 (18)	SD rats (20/20)	210–250 g	MCAO/2 h (ZL)	4% chloralhydrate	EA 30 min after reperfusion per day, with frequency of 5–10 HZ and current density of 2 mA (quchi, zusanli)	Non-EA	Proliferation	1. NFS (NSS, 3, 5, 7, 14d); 2. Brdu^+^ cell; 3. Nestin^+^ cell.	1. *P* < 0.05 (5, 7, 14d); 2. *P* < 0.05; 3. *P* < 0.05.
Wang 2013 (19)	SD rats (male, 12/12)	250–300 g	MCAO/2 h (ZL)^+^ transplantation of BMSC	4% chloralhydrate	EA 30 min after reperfusion per day, with frequency of 2–15HZ and current density of 1 mA (baihui, shuigou)	Non-EA^+^ transplantation of BMSC	Proliferation	1. NFS (mNSS, 7, 14d); 2. Infarct volume; 3. Brdu^+^ cell; 4. NSE^+^ cell.	1. *P* < 0.05; 2. *P* < 0.05 (14d); 3. *P > *0.05; 4. *P* < 0.01 (14d)
Tang 2008 (20)	Wistar rats (12/12)	250–300 g	MCAO	10% chloralhydrate (35 mg/Kg)	EA 15 min after surgery per day, with frequency of 5–10 HZ and current density of 2 mA (quchi, zusanli)	Non-EA	Differentiation	1. Dil/Brdu/NeuN^+^ cell (7, 14, 21d); 2. Dil/Brdu/GFAP^+^ cell	1. *P* < 0.05; 2. *P* < 0.05
Liu 2001 (21)	SD rats (male, 20/20)	250–280 g	Permanent MCAO	1% pentobarbital sodium	EA 30 min after surgery per day, with frequency of 5–10 HZ and current density of 1–5 mA (baihui, dazhui)	Non-EA	Proliferation	1. NFS (Bederson, 3, 7, 14, 21d); 2. Brdu^+^ cells.	1. *P* < 0.05; 2. *P* < 0.05
Tao 2008 (22)	SD rats (male, 27/27)	250 ± 30 g	MCAO/2 h (ZL)	10% chloralhydrate (0.3 ml/100 g)	EA 30 min after reperfusion per day, with frequency of 1–20 HZ and current density of 6 V (quchi, zusanli)	Non-EA	Proliferation, differentiation	1. Brdu^+^ cells (4, 7, 14d); 2. Brdu/NeuN^+^ cells; 3. Brdu/GFAP^+^ cells.	1. *P* < 0.05 (7d); 2. *P* > 0.05; 3. *P* < 0.05 (7d).
Diao 2008 (23)	Wistar rats (male, 23/26)	250 ± 10 g	MCAO/2 h (ZL)	10% chloralhydrate (350 mg/Kg)	EA 20 min after reperfusion per day, with frequency of 30–100 HZ and current density of 6–15 V (chengjiang, qihai, guanyuan)	Non-EA	Proliferation	1. Brdu^+^ cells (7, 14, 28d); 2. Brdu/Nestin^+^ cells.	1. *P* < ;0.01; 2. *P* < 0.05/0.01).
Yang 2006 (24)	Wistar rats (male, 18/18)	250 ± 20 g	MCAO/2 h	10% chloralhydrate (350 mg/Kg)	EA 10 min after reperfusion per day,(chengjiang, qihai, guanyuan)	Non-EA	Proliferation, differentiation	1. Brdu^+^ cells (7, 14, 28d); 2. Brdu/GFAP^+^ cells; 3. Brdu/Nestin^+^ cells.	1. *P* < 0.01 (14, 28d); 2. *P* < 0.01 (28d); 3. *P* < 0.05 (14, 28d).
Song 2008 (25)	SD rats (male, 12/12)	250–300 g	MCAO/30 min (Koizumi)	10% chloralhydrate (3 ml/Kg)	MA 20 min after reperfusion per day,(shuigou, neiguan, quchi, zusanli)	Non-MA	Differentiation.	1. NFS (Bederson, 2, 7d); 2. GFAP level.	1. *P* < ;0.05 (7d); 2. *P* < 0.01 (7d).
Yang 2008 (26)	Wistar rats (male, 30/30)	250 ± 10 g	MCAO/2 h	10% chloralhydrate (35 mg/100 g)	EA 20 min after reperfusion per day, with frequency of 30/100 Hz and current density of 5 V (Du: renzhong, baihui)	Non-EA	Differentiation.	GFAP^+^ cells (7, 14, 28d)	*P* < 0.05 (14, 28d)
					(Rendu: chengjiang, guanyuan, renzhong, baihui)			GFAP^+^ cells	*P* < 0.05 (14, 28d)
Pi 2006 (27)	Wistar rats (male, 22/24)	250 ± 10 g	MCAO/2 h + bFGF	10% chloralhydrate (35 mg/100 g)	EA 20 min after reperfusion per day, with frequency of 30/100 Hz and current density of 6–15 V (chengjiang, qihai, guanyuan)	Non-EA + bFGF	Proliferation	1. Brdu^+^ cells (7, 14, 28d); 2. Brdu/Nestin^+^ cells.	1. *P* < 0.05; 2. *P* < 0.05.
Ye 2012 (28)	Wistar rats (male, 60/60)	250 ± 30 g	MCAO	2% pentobarbital sodium (45 mg/Kg)	EA 15 min after surgery per day, with frequency of 5 Hz and current density of 2 mA, total 7 days (zusanli)	Non-EA	Proliferation	1. Brdu^+^ cells; 2. Nestin^+^ cells.	1. *P* < 0.05; 2. *P* < 0.05.
Li 2008 (29)	SD rats (male, 6/18)	220 ± 10 g	MCAO/2 h (ZL)	10% chloralhydrate (3 ml/Kg)	EA 30 min after reperfusion per day, with frequency of 2–15 Hz and current density of 1–5 mA, total 7 days (shuigou, baihui)	Non-EA	Proliferation	Brdu^+^ cells (7, 14, 30d)	*P* < 0.01 (7d)
You 2012 (30)	SD rats (male, 24/24)	450–500 g	Permanent MCAO	10% chloralhydrate (25 mg/100 g)	EA 20 min after surgery per day, with frequency of 2 Hz and current density of 1–2 mA (shousanli, waiguan, futu, zusanli)	Non-EA	Proliferation	1. NFS (Garcia, 1, 2, 3, 4w); 2. Infarct volume; 3. Nestin^+^ cells; 4. GAP-43^+^ cells.	1. *P* < 0.05 (2, 3d); 2. *P* < 0.05 (1, 2, 3w); 3. *P* < 0.05 (2, 3w); 4. *P* < 0.05 (2, 3w).
Mi 2010 (31)	SD rats (male, 90/90)	220–280 g	MCAO (ZL)	3.5% chloralhydrate (1 ml/100 g)	EA 20 min after surgery per day, with frequency of 10 Hz (baihui, quchi)	Non-EA	Proliferation	1. NFS (ZL, 6 h, 3, 7, 14, 21d); 2. Muscle testing; 3. GAP-43^+^ cells	1. *P* < 0.01 (21d), *P > *0.05 (6 h, 3, 7, 14d); 2. *P* < 0.01 (14, 21d); 3. *P* < 0.01 (3, 7, 14d).
Bao 2007 (32)	Wistar rats (10/10)	300 ± 20 g	MCAO/ 1.5 h (ZL)	10% chloralhydrate (3 ml/Kg)	EA 30 min after reperfusion per day, with frequency of 5–30 Hz and current density of 2 V, total 28 days (baihui, dazhui, guanyuan, housanli)	Non-EA nor MA	Proliferation, differentiation	1. NFS (ZL, 1–28d); 2. Brdu^+^ cells (28d); 3. Nestin^+^ cells; 4. GFAP mRNA level; 5. Nestin mRNA level.	1. *P* < 0.05 (14, 17, 21, 24, 28d); 2. *P* < 0.01; 3. *P* < 0.01; 4. *P* < 0.05; 5. *P* < 0.01.
					MA 30 min after reperfusion per day, with frequency of 5–30 Hz and current density of 2 V, total 28 days (baihui, dazhui, guanyuan, housanli)	Non-EA nor MA			1. *P* < 0.05 (14, 17, 21, 24, 28d); 2. *P* < 0.01; 3. *P* < 0.01; 4. *P* < 0.05; 5. *P* < 0.05.
Chen 2003 (33)	SD rats (male, 18/18)	200 ± 20 g	MCAO/2 h (ZL)	10% chloralhydrate (3 ml/Kg)	EA 30 min after reperfusion per day, with frequency of 4–16 Hz and current density of 3 mA (baihui, shangxing)	Non-EA;	Proliferation, migration.	1. NFS (ZL, 1–14d); 2. Infarct volume (4, 7, 14d); 3. Nestin^+^ cells; 4. Brdu^+^ cells; 5. PSA-NCAM.	1. *P* > 0.05; 2. *P* < 0.05 (7d), *P* > 0.05 (4, 14d); 3.*P* > 0.05; 4. *P* > 0.05; 5. *P* > 0.05.
Li 2004 (34)	SD rats (male, 40/40)	450–500 g	Permanent MCAO (Wahl)	10% chloralhydrate (300 mg/Kg)	EA 20 min after surgery per day, with frequency of 75 min^−1^ and current density of 1–2 mA (shousanli, waiguan, futu, zusanli)	Non-EA	Proliferation, migration, differentiation	1. NFS (Garcia, 1–14d); 2. Infarct size (1, 2, 3, 4w); 3. Brdu^+^ cells; 4. PSA-NCAM^+^ cells; 5. DCX^+^ cells; 6. Brdu/NeuN^+^ cells; 7. Brdu/GFAP^+^ cells	1. *P* < 0.05 (2, 3d); 2. *P* < 0.05 (2, 3w); 3. *P* < 0.05 (1, 2w); 4. *P* < 0.05 (1, 2, 3w); 5. *P* < 0.05; 6. *P* < 0.05 (1w); 7. *P* < 0.05 (3, 4w).
Zhang 2011 (35)	Wistar rats (female, 30/30)	200 ± 20 g	MCAO/1 h (ZL)	10% chloralhydrate (30 mg/Kg)	EA 20 min after reperfusion per day, with frequency of 2–100 Hz and current density of 2 mA	Non-EA	Proliferation, migration	1. NFS (ZL, 7, 14, 28d); 2. Brdu^+^ cells; 3. Brdu/ PSA-NCAM^+^ cells.	1. *P* < 0.05 (28d); 2. *P* < 0.01; 3. *P* < 0.01.
Zhao 2006 (36)	Wistar rats (male, 10/10)	320 ± 20 g	MCAO (ZL)	10% chloralhydrate (3 ml/Kg)	EA 15 min after surgery per day, with frequency of 5–30 Hz and current density of 0.8 mA, total 24 days. (baihui, fengfu)	Non-EA nor moxibustion	Proliferation, differentiation	1. NFS (ZL, 1–24d); 2. Nestin^+^ cells; 3. Brdu^+^ cells; 4. Nestin mRNA level; 5. GFAP mRNA level.	1. *P* < 0.05 (13, 16, 19, 22, 24d); 2. *P* < 0.01; 3. *P* < 0.01; 4. *P* < 0.05; 5. *P* < 0.05.
					Moxibustion 15 min after surgery per day, total 24 days. (baihui, guanyuan, zusanli)				1.*P* < 0.05 (13, 16, 19, 22, 24d); 2. *P* < 0.01; 3. *P* < 0.01; 4. *P* < 0.01; 5. *P* < 0.05
Liu 2005 (37)	SD rats (male, 36/36)	250–280 g	Permanent MCAO	10 g/l pentobarbital sodium (50 mg/Kg)	EA 30 min after surgery per day, with frequency of 5–10 Hz. (baihui, dazhui)	Non-EA	Proliferation	1. NFS (Bederson, 3, 7, 14, 21d); 2. Nestin^+^ cells.	1. *P* < 0.05/0.01; 2. *P* < 0.05/0.01.
Yu 2010 (38)	Wistar rats (male, 25/25)	360 ± 20 g	MCAO (ZL)	10% chloralhydrate (35 mg/Kg)	EA 30 min after surgery per day (baihui, dazhui)	Non-EA	Proliferation	1. NFS (ZL, 1, 3, 7, 14, 21d); 2. Nestin^+^ cells.	1. *P* < 0.05/0.01; 2. *P* < 0.05/0.01 (3, 7, 14, 21d).
Wan 2010 (39)	SD rats (male, 48/48)	90–120 g	MCAO/2 h (ZL)	NR	EA 15 min after reperfusion per day, with frequency of 3 Hz and current density of 3 V, total 14 days. (baihui, dazhui)	Non-EA	Differentiation.	1. BWC (1, 7, 14, 28d); 2.GFAP^+^ cells.	1. *P* < 0.05 (1, 7d); 1. *P* < 0.05 (7, 14, 28d).
Zhao 2004 (40)	Wistar rats (male, 40/40)	250–300 g	MCAO/1 h (ZL)	3.5% chloralhydrate (10 ml/Kg)	EA 15 min after reperfusion per day, with frequency of 60/40 Hz and current density of 1.5 V, total 7 days. (hegu-L14)	Non-EA	Proliferation	1. Infarct volume (1d); 2. NFS (ZL, 1, 7, 14, 21, 28d); 3.Brdu^+^ cells; 4.Nestin^+^ cells.	1. *P* < 0.05; 2. *P* < 0.05; 3.*P* < 0.05 (7d); 4. *P* < 0.05 (7d).
Tao 2010 (41)	SD rats (male, 20/20)	250 ± 30 g	MCAO/2 h (ZL)	2% isoflurane	EA 30 min after reperfusion per day, with frequency of 1–20 Hz (quchi, zusanli)	Non-EA	Proliferation, differentiation	1. NFS (Garcia, 4, 7, 14, 21d); 2.Brdu^+^ cells; 3. Brdu/GFAP^+^ cells; 4. Brdu/NeuN^+^ cells.	1. *P* < 0.05 (7, 14, 21d); 2.*P* < 0.05 (7, 14d); 3. *P* < 0.05 (7, 14d); 4. *P* < 0.05 (21d).
Yang 2005 (42)	SD rats (male, 6/8)	220–250 g	MCAO/ 30 min (ZL)	10% chloralhydrate (360 mg/Kg)	EA 20 min after reperfusion per day, with frequency of 60/2 Hz and current density of 10 mA, total 13 days. (fengfu, jinsuo)	Non-EA	Proliferation, differentiation	1. Infarct volume; 2. Brdu^+^ cells; 3. Brdu/CRMP-4^+^ cells; 4. Brdu/MAP-2^+^ cells	1. *P* < 0.05; 2. *P* < 0.05; 3. *P* < 0.05; 4. *P* < 0.05.
Cheng 2009 (43)	SD rats (male, 12/12)	250–280 g	MCAO/2 h + PBS	10% chloralhydrate (350 mg/Kg)	EA 30 min after reperfusion per day, with frequency of 4 Hz and current density of 2 mA, total 3 days. (baihui, renzhong)	Non-EA + PBS	Proliferation	1. NFS (mNSS, 1, 7, 14, 21, 28d); 2. Infarct volume (28d); 3. Brdu^+^ cells (7, 28d).	1. *P* > 0.05; 2. *P* > 0.05; 3. *P* < 0.05.
Kim 2014 (44)	C57BL/6 mice (male, 6/6)	NR	MCAO/ 40 min (ZL)	isoflurane	EA 20 min from 5 day after reperfusion per day, with frequency of 2 Hz and output voltage of 2 V, total 10 days. (baihui GV20, dazhui GV14)	Non-EA	Proliferation, Differentiation	1. Rotarod test (40, 47d); 2. Morris water maze tests (44–46d); 3. Brdu^+^ cells (14d); 4. Brdu/Dcx^+^ cells (14d); 5. Brdu/NeuN^+^ cells (47d); 6. Brdu/ GFAP^+^ cells (47d).	1. *P* < 0.05/0.01; 2. *P* < 0.05; 3. *P* < 0.01; 4. *P* < 0.01; 5. *P* < 0.05; 6. *P* < 0.001.

Note: TMS (total motor scores; CB: calbindin; MCAT: Middle CerebralArtery Trumbosis; PCNA proliferation cell nuclear antigen; BWC: Brain water content; NFS: Neural functional score; BMSC: bone marrow-derived mesenchymal stem cells; EA: electroaucpunture; MA: manual acupuncture; PSA-NCAM: polysialylated form of the neural cell adhesion molecule; Brdu: bromodeoxyuridine; GFAP: Glial fibrillary acidic protein; NR: no report.

**Table 2:** Risk of bias of the included studies.

Study	A	B	C	D	E	F	G	H	I	J	Total
Huang 2009 (11)	√	√	√			√			√	√	**6**
Yan 2008 (12)	√	√	√			√			√		**5**
Ren 2007 (13)	√		√			√	√		√	√	**6**
Gao 2010 (14)	√						√		√	√	**4**
Luo 2007 (15)		√	√			√			√	√	**5**
Yang 2010 (16)	√		√	√							**3**
Rui 2011 (17)	√		√			√			√	√	**5**
Li 2012 (18)	√		√			√					**3**
Wang 2013 (19)	√		√			√					**3**
Tang 2008 (20)	√		√			√					**3**
Liu 2001 (21)	√		√			√					**3**
Tao 2008 (22)	√	√	√			√					**4**
Diao 2008 (23)	√	√	√			√			√		**5**
Yang 2006 (24)	√		√			√			√		**4**
Song 2008 (25)	√	√	√			√			√		**5**
Yang 2008 (26)	√	√	√			√			√		**5**
Pi 2006 (27)	√		√			√			√		**4**
Ye 2012 (28)	√		√			√					**3**
Li 2008 (29)		√	√			√			√		**4**
You 2012 (30)		√	√			√	√		√	√	**6**
Mi 2010 (31)		√	√			√			√	√	**5**
Bao 2007 (32)		√	√			√				√	**4**
Chen 2003 (33)		√	√			√				√	**4**
Li 2004 (34)	√	√	√			√	√			√	**6**
Zhang 2011 (35)	√	√	√			√			√		**5**
Zhao 2006 (36)		√	√			√			√	√	**5**
Liu 2005 (37)	√		√			√			√		**4**
Yu 2010 (38)	√	√	√			√			√		**5**
Wan 2010 (39)	√		√			√			√		**4**
Zhao 2004 (40)	√		√			√			√	√	**5**
Tao 2010 (41)	√	√	√			√			√	√	**6**
Yang 2005 (42)	√		√			√			√	√	**5**
Cheng 2009 (43)	√	√	√			√			√	√	**6**
Kim 2014 (44)	√	√				√			√	√	**5**

Note: Studies fulfilling the criteria of: A: peer reviewed publication; B: control of temperature; C: random allocation to treatment or control; D: blinded induction of model; E: blinded assessment of outcome; F: use of anesthetic without significant intrinsic neuroprotective activity; G: appropriate animal model (aged, diabetic, or hypertensive); H: sample size calculation; I: compliance with animal welfare regulations; J: statement of potential conflict of interests.

**Table 3:** Subgroup analyses for the effect of acupuncture on neurogenesis after ischemic stroke.

Brdu (7d)			Nestin (7d)				PSA-NCAM (7d)				NeuN (14d)			
Groups	n	MD [95%CI]	*P*	Ad R^2^(%)	n	MD [95%CI]	*P*	Ad R^2^(%)	n	MD [95%CI]	*P*	n	MD [95%CI]	*P*
All trials	19	32.85 [20.78,44.91]			16	37.39 [24.69, 50.09]			6	30.90 [17.39, 44.40]		6	22.62 [15.16, 30.07]	
Encephalic region			<0.001	N/A			<0.001	1.21			0.15			<0.001
SVZ	10	31.64 [30.61, 32.67]			9	56.82 [17.75, 95.89]			3	61.72 [21.82, 101.61]		3	4.25 [3.34, 5.17]	
SGZ	9	11.11 [10.42, 11.79]			6	13.18 [7.65, 18.71]			5	30.42 [16.12, 44.72]		3	10.26 [9.02, 11.51]	
Species			0.01	14.33			0.006	1.99			<0.001			0.22
SD rats	10	18.80 [12.11, 25.50]			6	11.70 [6.64, 16.77]			5	1.41 [0.14, 2.67]		3	6.77 [5.77, 7.76]	
Wistar rats	9	48.23 [26.68, 69.78]			10	48.01 [22.56, 73.47]			3	17.54 [14.27, 20.81]		3	5.85 [4.75, 6.95]	
MCAO			<0.001	23.17			0.14	N/A			<0.001			<0.001
Permanent	4	7.79 [4.06, 11.52]			4	19.38 [11.69, 27.08]			2	9.29 [2.02, 16.57]		1	1.07 −1.39, 3.53]	
Transient	14	36.01 [24.63, 47.38]			9	33.10 [13.99, 52.21]			4	44.19 [26.11, 62.26]		4	37.50 [23.29, 51.71]	
NR	1	89.50 [87.14, 91.86]			3	61.65 [8.51, 114.78]			/	/		/	/	
Anesthetic			<0.001	N/A			<0.001	6.22			N/A			N/A
Chloralhydrate	16	13.73 [13.09, 14.37]			12	11.43 [10.45, 12.41]			6	30.90 [17.39, 44.40]		6	22.62 [15.16, 30.07]	
Pentobarbital sodium	3	31.28 [30.04, 32.52]			2	17.98 [16.66, 19.30]			/	/		/	/	
NR	/	/			2	15.84 [14.41, 17.28]			/	/		/	/	
Weight (gram)			0.79	N/A			0.001	N/A			0.11			0.001
> 300	3	40.02 −20.62, 100.67]			2	13.06 [10.92, 15.20]			2	20.32 [11.49, 29.15]		2	6.35 [2.97, 9.73]	
< = 300	16	31.51 [18.50, 44.51]			12	45.40 [26.02, 64.78]			4	36.07 [19.18, 52.96]		4	22.28 [13.17, 31.38]	
NR	/	/			2	15.84 [14.41, 17.28]			/	/		/	/	
Duration of treatment (min)			<0.001	31.16			<0.001	7.70			N/A			<0.001
> 15	14	32.85 [20.78, 44.91]			10	8.18 [7.37, 9.00]			6	30.90 [17.39, 44.40]			3.56 [2.37, 4.75]	
< = 15	5	64.82 [45.12, 84.53]			6	29.46 [28.17, 30.76]			/	/			5.33 [4.68, 5.99]	
Baseline			0.01	24.43			0.02	48.78			0.01			N/A
mean > 100	11	44.17 [23.32, 65.01]			4	95.65 [31.27, 160.04]			3	59.03 [21.78, 96.29]		6	2.44 [1.23, 3.65]	
mean < = 100	8	16.21 [10.56, 21.85]			12	17.35 [12.91, 21.79]			3	10.00 [−1.67, 21.67]		/	/	

*P* value for test for subgroup differences. Adjusted R^2^ for study characteristics accounting for heterogeneity. Ad, adjusted; MCAO, middle cerebral artery occlusion; N/A: not acquired; NR, no report; SGZ, subgranular zone; SVZ, subventricular zone.
