# Supplementary material for: Acupuncture for neurogenesis in experimental ischemic stroke: a systematic review and meta-analysis
Source: Sci Rep. 2016 Jan 20;6:19521. doi: 10.1038/srep19521 (PMC4726177; doi:10.1038/srep19521)
Supplement: Supplementary Information [file srep19521-s1.pdf]

Supplementary Information

Acupuncture for neurogenesis in experimental ischemic stroke: a systematic review and meta-analysis

Lin Lu[1, 2], Xiao-guang Zhang[2], Linda LD Zhong[1], Zi-xian Chen[2],Yan Li [2], Guo-qing Zheng[2] & Zhao-xiang Bian[1]

1 School of Chinese Medicine, Hong Kong Baptist University, Hong Kong SAR, China.
2 Department of Neurology, the Second Affiliated Hospital and Yuying Children's Hospital of Wenzhou Medical University, Wenzhou 325027, China.

The Supplementary Information consists of one PDF document containing Figures S1-S5,

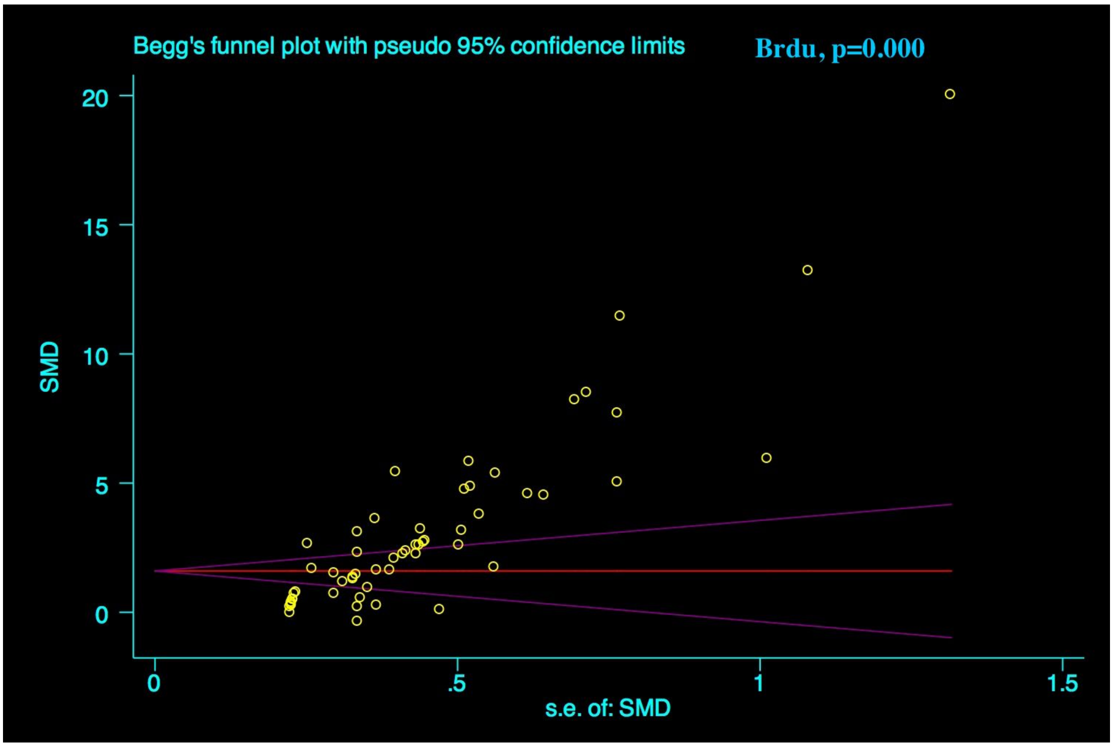

Figure S1: Linear regression analysis of the funnel plot of acupuncture for Brdu marker of neurogenesis in experimental ischemic stroke (Egger's test, p=0.000).

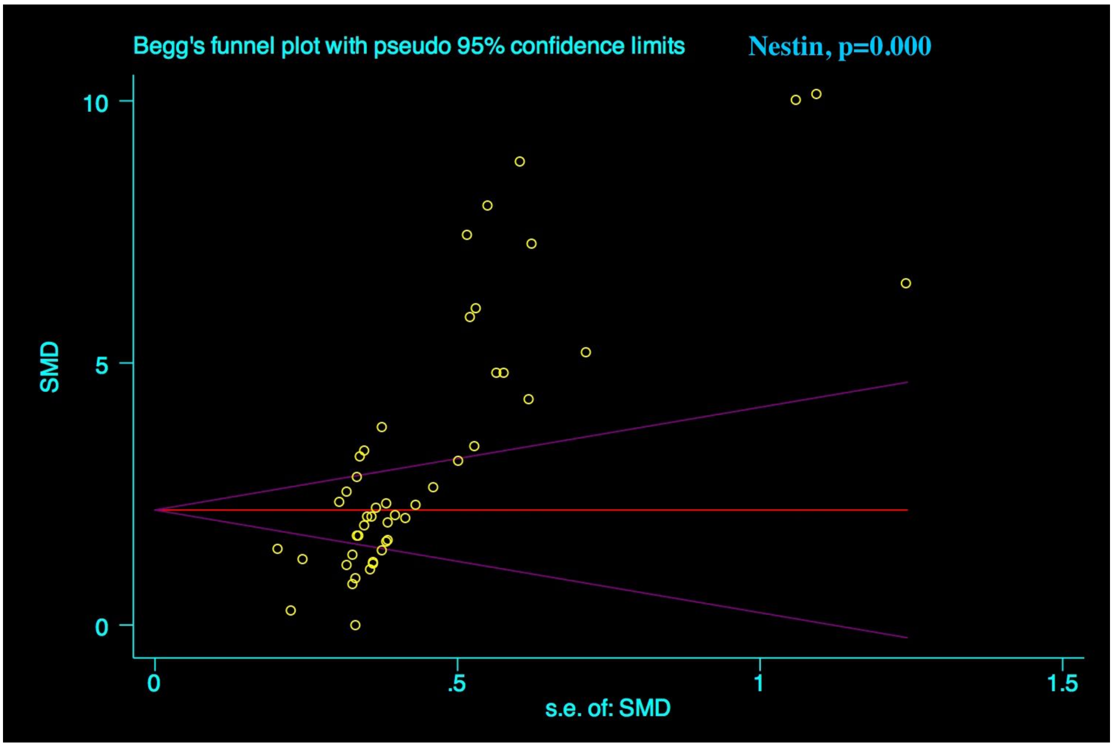

Figure S2: Linear regression analysis of the funnel plot of acupuncture for Nestin marker of neurogenesis in experimental ischemic stroke (Egger's test, p=0.000).

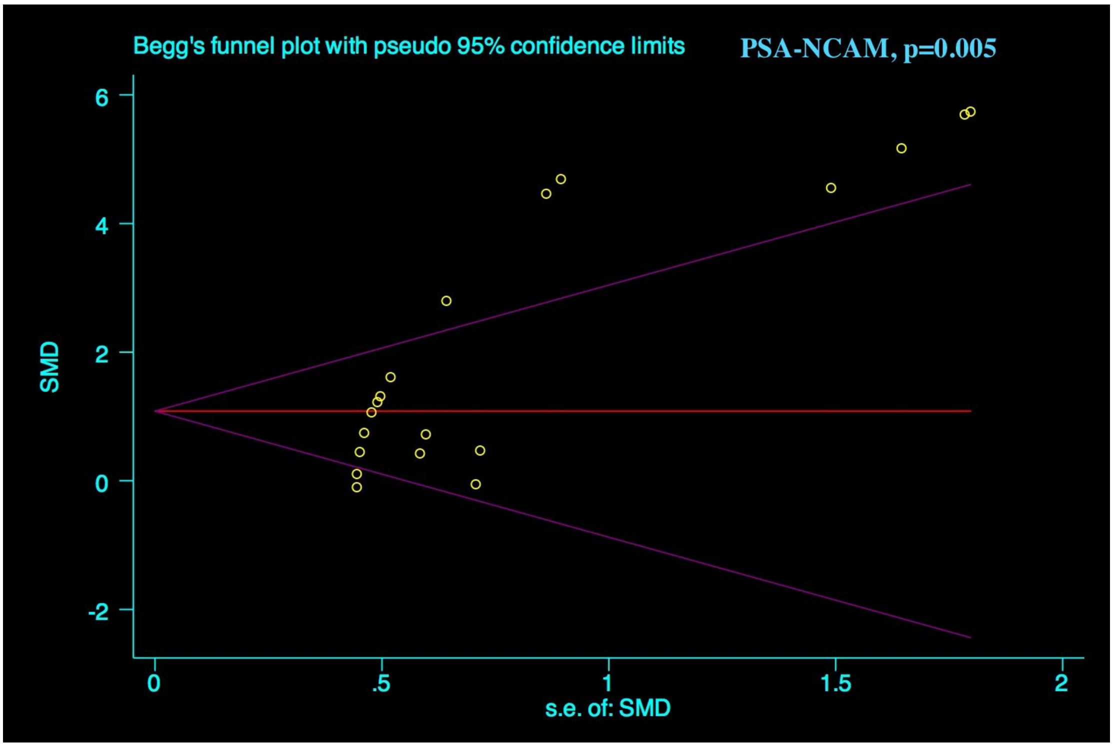

Figure S3: Linear regression analysis of the funnel plot of acupuncture for PSA-NCAM marker of neurogenesis in experimental ischemic stroke (Egger's test, p=0.005).

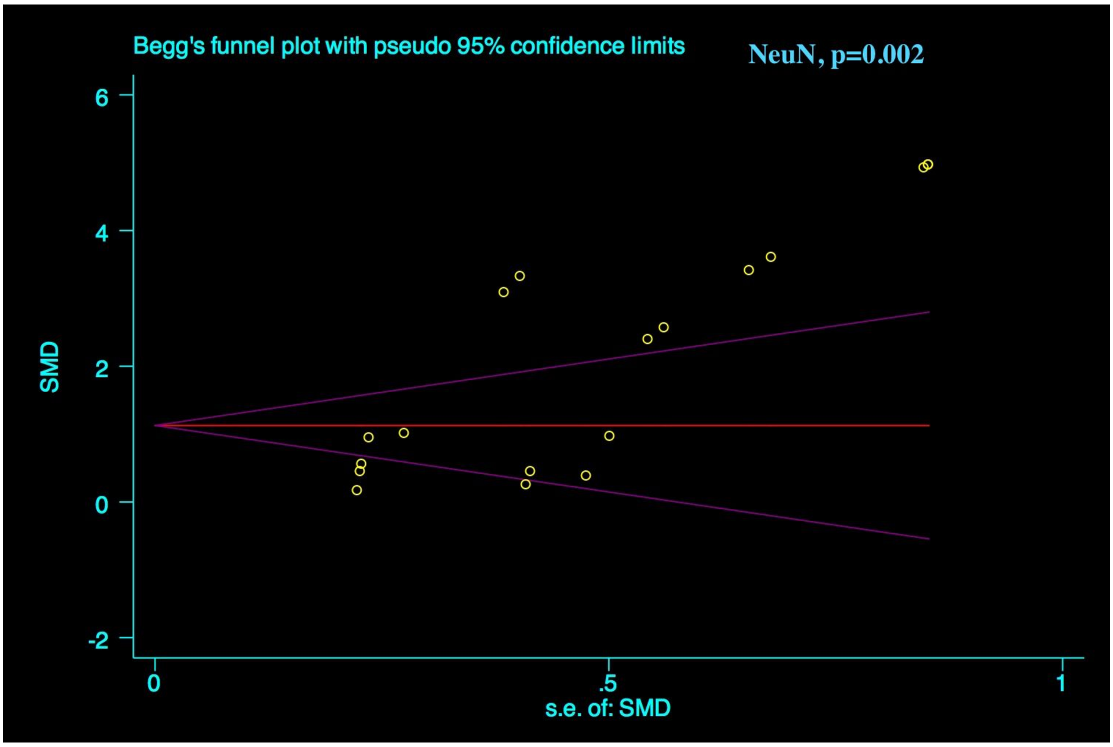

Figure S4: Linear regression analysis of the funnel plot of acupuncture for NeuN marker of neurogenesis in experimental ischemic stroke (Egger's test, p=0.002).

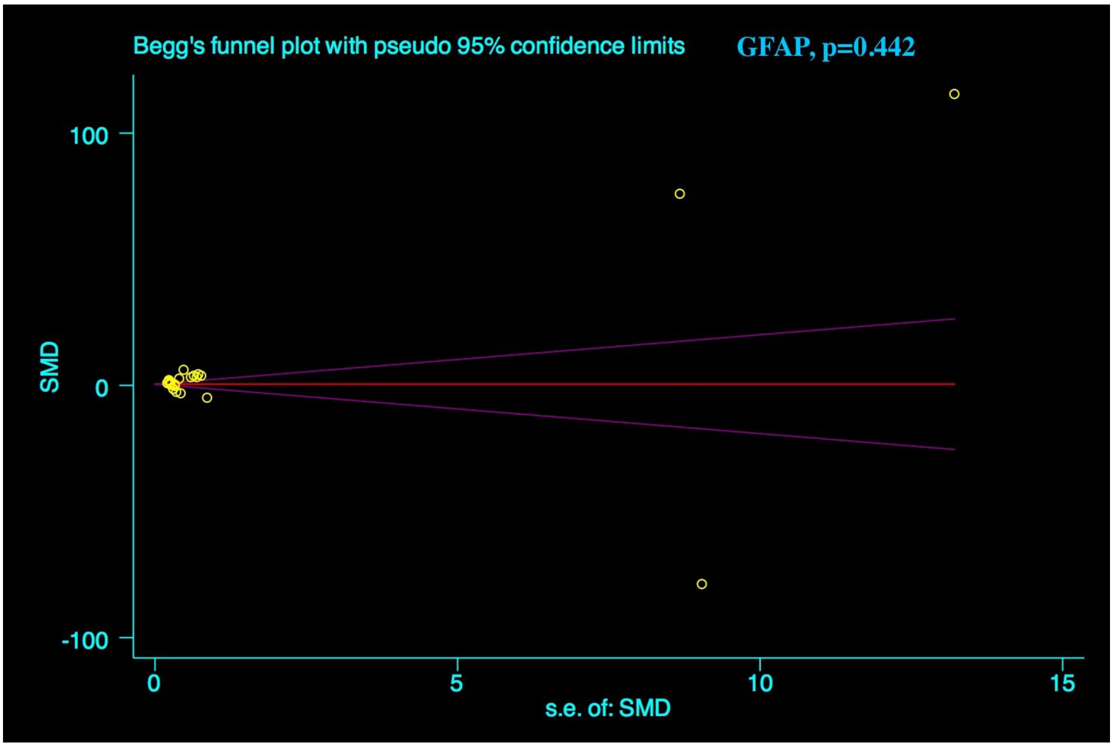

Figure S5: Linear regression analysis of the funnel plot of acupuncture for GFAP marker of neurogenesis in experimental ischemic stroke (Egger's test, p=0.442).
